# Herbal Compounds and Toxins Modulating TRP Channels

**DOI:** 10.2174/157015908783769644

**Published:** 2008-03

**Authors:** Joris Vriens, Bernd Nilius, Rudi Vennekens

**Affiliations:** Laboratory of Ion Channel Research, Department of Mol. Cell Biology, Division of Physiology, Campus Gasthuisberg, KU Leuven, Herestraat 49, B-3000 LEUVEN, Belgium

## Abstract

Although the benefits are sometimes obvious, traditional or herbal medicine is regarded with skepticism, because the mechanism through which plant compounds exert their powers are largely elusive. Recent studies have shown however that many of these plant compounds interact with specific ion channels and thereby modulate the sensing mechanism of the human body. Especially members of the Transient Receptor Potential (TRP) channels have drawn large attention lately as the receptors for plant-derived compounds such as capsaicin and menthol. TRP channels constitute a large and diverse family of channel proteins that can serve as versatile sensors that allow individual cells and entire organisms to detect changes in their environment. For this family, a striking number of empirical views have turned into mechanism-based actions of natural compounds. In this review we will give an overview of herbal compounds and toxins, which modulate TRP channels.

## INTRODUCTION

Finding healing powers in plants is an ancient idea. People in all continents have long used hundreds, if not thousands, of indigenous plants for treatment of various ailments dating back to prehistory. Over the years, natural products have contributed enormously to the development of important therapeutic drugs used currently in modern medicine. Besides the clinical use of natural products or their compounds to treat diseases, these substances have also been important tools for the discovery of new targets such as receptors or ion channels. Exemplary in this regard is the *t*ransient *r*eceptor *p*otential (TRP) family of ion channels.

At the moment more than 50 members of the TRP family have been characterized in yeast, worms, insects, fish and mammals [84, 127]. Based on structure homology, TRP channels can be classified into seven subfamilies TRPC (canonical or classical), TRPV (vanilloid), TRPM (melastatin), TRPA (ANKTM1 homologues), TRPP (polycystin), TRPML (mucolipin), and TRPN (NOMP-C homologues) [21, 127]. TRP channels are intrinsic membrane proteins with six putative transmembrane spans (TM) and a cation-permeable pore region formed by a short hydrophobic stretch between TM5 and TM6. For a detailed overview of the structural differences of the different subfamilies, we refer to some recent reviews [81, 88, 90, 124]. The known functions of TRP channels are diverse. Nematodes use TRP channels at the tips of neuronal dendrites in their ‘noses’ to detect and avoid noxious chemicals. Humans use distinct TRP channels to appreciate sweet, bitter and umami tastes, and warmth, heat and cold [25]. TRP channels can serve as versatile sensors that allow individual cells and entire organisms to detect changes in their environment [124]. In fact, members of the TRP family are at the vanguard of our sensory systems, responding to temperature, touch, pain, osmolarity, pheromones, taste and other stimuli.

In this review we show that many TRP channels are chemesthetic receptors. Since several naturally occurring substances are able to modulate/ interact with these sensing channels, they provide a range of new potential targets for the development of therapeutic drugs.

## TRPC CHANNELS

Members of the TRPC subfamily are closely related to *Drosophila* TRP (30-40% identity) [127]. The TRPC subfamily is readily divided into three groups on the basis of sequence alignments and functional comparisons: TRPC1/ 4/5, TRPC3/6/7, and TRPC2 [21]. 

Recently TRPC1 and TRPC6 were reported to be mechanically activated cation channels [67, 101]. The stretch response of TRPC6 is blocked by the tarantula peptide, GsMTx-4 [101]. Whether TRPC1 is also blocked by this toxin is a matter of debate at the moment [11]. The peptide GsMTx-4 is isolated from the venom of the Chilean Rose tarantula, *Grammostola spatulata*, and is the only drug known to specifically affect cationic stretch-activated-ion channels (for a review see [11]). Structurally, GsMTx-4 follows the inhibitory cysteine knot motif with six cysteines and is amphipathic. The hydrophobic surface is surrounded by mostly positive charges. This design suggests that the peptide binds to membranes using its hydrophobic face to penetrate the lipid bilayer. The response of TRPC6 to diacylglycerol (DAG) is also inhibited by GsMTx-4, suggesting that DAG is probably acting as an amphipath, creating stress in the boundary layer and thereby activating TRPC6 [101].

Recently it was shown that TRPC6 is activated by a key constituent of St. John’s wort, i.e. hyperforin [55]. St. John’s worth is traditionally used as a herbal anti-depressant, an effect which can be largely ascribed to hyperforin interfering with the neuronal uptake of serotonin, dopamine and norepinephrine and the inhibition of GABA and L-glutamate uptake [74].

## TRPV CHANNELS

The TRPV subfamily contains six members in vertebrates, and is divided in two groups: TRPV1-V4 and TRPV5-V6 [21]. TRPV1, TRPV2, TRPV3 and TRPV4 are Ca^2+^–permeable, nonselective cation channels that can be activated by heating in heterologous expression systems. TRPV5 and TRPV6 are highly Ca^2+^-selective channels with low temperature sensitivity [20, 85]. TRPV1-4 channels are expressed in sensory neurons, mediate nociception and contribute to the detection and integration of diverse (including thermal) stimuli [124]. 

## TRPV1

The fruits of *Capsicum* plants are commonly known as chili peppers and their use in culinary preparations and in traditional medicine is known since many centuries. The traditional medical uses of *Capsicum* include appetite stimulation, treatment of gastric ulcers and rheumatism, restoration of hair growth, and the relief of toothache [112]. Since capsaicin exhibited selectivity for a subset of sensory neurons, an early suggestion was that certain sensory neurons might express a specific receptor for this natural substance. Subsequently the TRPV1 receptor was identified in an expression cloning experiment using capsaicin [17]. Further characterization of TRPV1 showed predominant expression in sensory neurons [17]. The channel responds to noxious stimuli, including capsaicin, heat, and acid [119]. TRPV1 is thought to play a crucial role in temperature sensing and nociception [15] and has attracted attention as a molecular target for pain treatment [109, 113]. 

### Capsaicinoids

Capsiate is a nonpungent capsaicin analog, obtained from a non-pungent cultivar of red peppers (as *Capsicum annuum*). The major pungent components in fruits of *Capsicum* plants are capsaicin, (*E*)-*N*-[(4-hydroxy-3-methoxyphenyl)methyl]-8-methyl-6-nonenamide, and dihydrocapsaicin, the structure of which is a 6,7-dihydro derivative of capsaicin. More than 12 other capsaicinoids have been found as minor components [106]. Several studies have reported on the pungency and bioactivity of various capsaicinoids. Watanabe *et al*. reported that several nonpungent capsaicinoids enhance adrenal catecholamine secretion as well as capsaicin. These nonpungent capsaicinoids are interesting from the viewpoint of wide application to foods and drugs [131]. There is evidence obtained from *in vivo* and *in vitro* studies that capsiate has similar effects as capsaicin [41]. The non-pungency of capsiate has been related with its poor accessibility to sensory neurons when administered to skin or mucosa by its degradation and trapping in the lipid phase of epithelium or cornea due to its high lipophilicity [41].

Piperine is an alkaloid found naturally in plants belonging to the *Piperaceae* family, such as *Piper nigrum*, commonly known as black pepper, and *Piper longum*, also known as long pepper. Black pepper has been used in traditional Chinese medicine to treat seizure disorders. It has putative anti-inflammatory activity and may have activity in promoting digestive processes. The first evidence for the action of piperine as a TRPV1 agonist was demonstrated by its ability to inhibit the binding sites of [^3^H]-RTX in the dorsal horn of pig spinal cord [115]. In addition, similar to capsaicin, piperine activates inward currents in TG neurons, an action that was found to be sensitive to capsazepine [58]. In a recent study, Gunthorpe and co-workers conclude that piperine is not only similar to capsaicin in its effects at human TRPV1 expressed in HEK293 cells but it also exhibits a greater efficacy than capsaicin itself for both activation and desensitization of TRPV1 [69]. At present, it is not clear why piperine exhibits an improved desensitization-to-excitation ratio compared with capsaicin. Structurally, the major difference between capsaicin and piperine is the replacement in piperine of the vanillyl moiety by a methylendioxy group, a known inhibitor of cytochrome P450 metabolism [95]. Alternatively, piperine might be more effective than capsaicin in promoting the dephosphorylated (inactive) state in TRPV1. Either way, this finding raises the possibility that piperine might serve as a chemical template for the design and synthesis of improved TRPV1 agonists.

Eugenol is an allyl chain-substituted guaiacol, i.e. 2-methoxy-4-(2-propenyl)phenol and is a member of the allylbenzene class of chemical compounds. Eugenol is the chief constituent of clove or clocimum oil obtained form *Eugenia carophyllata* and *Ocimum gratissimum*. After isolation of eugenol from clove oil, it was demonstrated in electrophysiological studies, that eugenol is able to activate inward currents in hTRPV1-HEK293 cells and TG neurons. This effect was completely prevented by capsazepine, indicating an action at the TRPV1 receptor [137]. *In vivo* experiments have shown that eugenol possesses anti-nociceptive properties that are somewhat comparable to that of capsaicin with its effect being reversed by capsazepine pre-treatment [137]. However, other TRP channels could also be activated by eugenol (see below).

### Resiniferanoids

The extremely irritant diterpene present in the dried latex of the plant *Euphorbia resinifera*, resiniferatoxin (RTX) is a very specific agonist for the TRPV1 channel. *E. resinifera* is a cactus-like plant native to the Anti-Atlas Mountains of Morocco [35]. Early reports of the medical use of dried latex of *E. resinifera* describe its direct application to dental cavities to mitigate toothache or to suppress chronic pain [3]. Because capsaicin and RTX analogues share a homovanillyl group as a structural feature motif essential for bioactivity, these naturally occurring substances were collectively called vanilloids. Importantly, RTX is about 3 to 4 magnitude more potent than capsaicin as well in dose-response curve as for the effect on thermoregulation and neurogenic inflammation [111].

### Ginger-Derived Products

These classes of compounds were isolated from ginger plants (*Zingiber officinale*), a reed-like plant. Ginger though called a root, is actually the rhizome of the monocotyledonous perennial plant *Zingiber officinale*. Ginger contains up to 3% of an essential oil that causes the fragrance of the spice. The main constituents are gingerols, such as [8]- and [6]- gingerol, other pungent substances, are shogaol, zingerone, and paradol. 

Gingerols ([6,8,10]-gingerols) and shogaols ([6,8,10]-shogaols) have differences in the length of the alkyl carbon chain. All different gingerols and shogaols increased intracellular calcium concentration in rat TRPV1-expressing HEK293 cells. In this regard, the shogaols were more potent than the gingerols [43]. Both [6]-gingerol and [8]-gingerol evoke capsaicin-like Ca^2+^ transients and ion currents in DRG neurons, with both effects being sensitive to the action of capsazepine [26]. Aversive responses were induced by [6]-, [10]-gingerol, and [6]-shogaol in rats when these compounds were applied to the eye; however, no response was observed in response to [10]-shogaol. [10]-Shogaol induced nociceptive responses via TRPV1 in rats following its subcutaneous injection into the hindpaw. In conclusion, gingerols and shogaols are both activators of the TRPV1 channel. Interestingly, [10]-shogaol is the only nonpungent compound among the gingerols and shogaols, suggesting its usefulness as a functional ingredient in food [43].

Zingerone is similar in chemical structure to other flavor chemicals such as vanillin and eugenol. It is used as a flavor additive in spice oils and in perfumery to introduce spicy aromas. Fresh ginger does not contain zingerone; cooking the ginger transforms gingerol, which is present, into zinge-rone. Out of experimental data, it has been suggested that capsaicin and zingerone could activate the same receptor. Cultured rat TG neurons and TRPV1-*Xenopus* oocytes were desensitized by repeated applications of zingerone [59]. Moreover, analysis in rTRPV1-HEK 293 cells showed that gingerols increased intracellular Ca^2+^ [133]. In this way, zingerone and gingerols represents a class of naturally occurring TRPV1 receptor agonists that might account for the medical properties of ginger, which have been known for centuries.

Paradol can be obtained from gingerol by successive dehydration and hydrogenation and is also found in the seeds of *Aframomum melegueta* as a major pungent principle. [6]-Paradol has been cited as pungent [53], but little is known about its possible direct activation of TRPV1.

### Ginsenosides

Ginsenosides are a class of steroid-like compounds, triterpene saponins, found exclusively in the plant genus *Panax* (ginseng), genus of about five or six species of slow-growing perennial plants with fleshy roots. The botanical name *Panax* means "all-heal" in Greek, and was aware of its wide use in Chinese medicine (for review see [14]). The major bioactive components of ginseng are ginsenosides. [139]. Ginsenosides can alleviate pain from injections of noxious chemicals, such as capsaicin [77]. Furthermore, there is evidence indicating that ginsenosides can directly block capsaicin-activated channels, resulting in attenuation of the currents in rat sensory neurons [32]. On the other hand, ginsenosides enhance the capsaicin-induced current in a concentration-dependent and reversible manner, but ginsenosides itself elicited no membrane currents in TRPV1-*Xenopus* oocytes [47]. However, the present results provide no concrete answers to the question about how the *in vitro* enhancement of capsaicin-induced currents by ginsenosides is related to the *in vivo* suppression of the capsaicin-induced pain related-behavior.

### Cannabinoids

Cannabinoids are a group of chemicals that referred to an unique group of secondary metabolites found in the cannabis plant, *Cannabis sativa*, which are responsible for the peculiar pharmacological effects of the plant. *Cannabis* species contain a complex mixture of substances that include 60 different cannabinoids, of whom tetrahydrocannabinol (THC), canna-bidiol (CBD) and cannabinol (CBN) are the most prevalent. Cannabinol is the primary product of tetrahydrocannabinol degradation, and there is usually little of it in a fresh plant. CBN content increases as THC degrades in storage, and with exposure to light and air. It is only mildly psychoactive, and is perceived to be sedative or stupefying. The biological actions of cannabidiol cannot be exclusively related to its cannabinoid receptor interaction, considering its low affinity (micromolar range) for cannabinoid receptors [10]. Interes-tingly, some actions of cannabidiol are similar to those of capsaicin, including anti-inflammatory and analgesic effects [61, 66]. More indications for a behavior as a full agonist of TRPV1 were given by Bisogno *et al.*, who showed that cannabidiol inhibits the binding of [^3^H]-RTX, and increase intracellular free Ca^2+^ in hTRPV1-HEK293 cells at micro-molar concentration [10]. Such interaction with TRPV1 seems to be related to some *in vivo* effects of cannabidiol, since it was shown that the TRPV1 receptor mediates the analgesic action of cannabidiol [22].

### Evodia Compounds

Evodiamine is a chemical which is extracted from fruits of *Evodia rutaecarpa.*Literature refers to *Evodia* fruits as a “hot nature” herb. In traditional Chinese, and Japanese medi-cine *Evodia* fruits have been prescribed for the treatment of headache, thoraco-abdominal pain, and vomiting that are caused by cold temperatures. Its mode of action is believed to be similar to that of capsaicin, since evodiamine is indeed a genuine agonist of TRPV1. It produces extracellular Ca^2+^ uptake as well as intracellular Ca^2+^ increase in rat TRPV1 expressing CHO cells, and both effects are competitively antagonized by capsazepine, a capsaicin antagonist and a blocker of TRPV1 [89]. The *in vivo* effects of evodiamine on sensory neurons demonstrate that evodiamine both activates and desensitizes the capsaicin-sensitive sensory afferents in mice, resulting in nociceptive and anti-nociceptive effects. The nociceptive action (paw licking behaviour) was dose dependently induced by intradermal injection of evodiamine and was suppressed by the co-treatment with capsazepine. The treatment with higher dosages of evodiamine showed sustained anti-nociceptive effects [49]. Evodiamine shows the analgesic action by desensitizing sensory nerves. It should be mentioned however that evodiamine doesn’t taste hot [50], indicating that it’s above mentioned effects might be mediated through it’s interaction with another factor other than the TRPV1 channel.

### Unsaturated 1,4-Dialdehydes Terpenes

To date, approximately 80 terpenoids containing an α,β-unsaturated 1,4 dialdehyde (3-formyl-3-butenal) functionality have been isolated from natural sources: the majority have been isolated from terrestrial plants and fungi, but also algae, liverworts, arthropods, sponges and mollusks. In general these compounds are believed to protect the producing organism from parasites and predators. However, it should be noted that several biological effects of unsaturated dialdehydes are of pharmacological interest (for example, terpenoid unsaturated dialdehydes have shown to reduce blood cholesterol and to inhibit platelet aggregation (for review see [103]).

Polygodial and drimanial are unsaturated 1,4-dialdehyde sesquiterpenes isolated from the bark of *Drymis winteri*, a native Brazilian medical plant used in folk medicine for the treatment of various inflammatory diseases, as well as for culinary substitution of pepper. Similar to capsaicin, systemic administration of polygodial and drimanial produces marked anti-nociceptive, anti-inflammatory and anti-allergic effects [70, 71, 98]. In addition, polygodial and drimanial displaced the specific binding of [^3^H]-RTX to rat spinal cord membranes. Likewise, same unsaturated 1,4-dialdehydes promoted an increase of [^45^Ca^2+^] uptake in rat spinal cord synaptosomes and increase the intracellular Ca^2+^ levels in cultured rat trigeminal neurons [2].

Isovelleral is a fungal sesquiterpene dialdehydes isolated from the large fungus, *Lactarius vellereus*that is considered inedible because of its peppery taste. Results suggest that isovelleral-like compounds produce their irritant effect by interacting with vanilloid receptors on capsaicin-sensitive sensory neurons [114], although later studies showed some contradictions about the effects of isovelleral [94]. 

Other unsaturated 1,4-dialdehydes terpenes are cinnamodial, cinnamosmolide, and cinnamolide present in the hot-tasting bark of *Cinnamosma fragrans*. This tree**is used traditionally as a decoction for treatment of malarial symptoms. These compounds are also capable of inducing Ca^2+^ uptake in rat DRG and inhibit specific [^3^H]-RTX binding site in rat spinal cord membranes [110]. Remarkably, at low concentrations, cinnamodial and cinnamosmolide evoke Ca^2+^ uptake in a concentration-dependent manner, while at higher concentrations these compounds caused blockade of Ca^2+^ uptake. Another prominent representative of the unsaturated 1,4-dialdehyde terpenes is warburganal, isolated from East African *Warburgia* plants, which shows a strong antifungal activity. Warburganal protects warburgia trees from the larvae of the African armyworm by interfering with their stimulus transduction process. Warburganal is also pungent, a property which might help to repel herbivores (for review see [103]). In fact, warburganal is a hot-tasting compound and activates capsaicin-sensitive nerves. Evidence for a role as TRPV1 agonist come from experiments indicating that this unique dialdehyde sesquiterpene inhibits specific [^3^H]-RTX binding sites in rat spinal cord preparations and induces pungency on the human tongue [114]. Other unsaturated dialdehyde sesquiterpenes like scalaradial (a marine natural product produced by sponges with anti-inflammatory activity), afromodial (derived from the plant *Aframomum danielli*), ancistrodial (the defensive sesquiterpene from *Ancistrotermes cavithorax*), merulidial (isolated from the culture fluid of the *Basidiomycete Merulius tremellosus*) and drimenol (from *Lactarius uvidus*) all inhibit [^3^H]-RTX binding sites in rat spinal cord preparations. Taken together, these findings suggest that part of the actions of these compounds could be related to their ability to activate the TRPV1 channel [110, 114]. Unsaturated 1,4-dialdehydes play a pivotal role in the chemical self-defence system of their host organisms.

### Triprenyl Phenols

Several prenylphenols from basidiocarps of European and Chinese *Albatrellus*, namely grifolin, neogrifolin, con-fluentin, scutigeral and albaconol seems to modulated the vanilloid receptor. Scutigeral was found to cause desensiti-zation of the capsaicin response, either in the presence or absence of external Ca^2+^. Grifolin, neogrifolin, and albaconol had been reported to antagonise the capsaicin-induced calcium response in a dose-dependent manner. In contrast to previous studies reported in the literature [112], latest data suggest that fungal prenylphenols act as weak antagonists (activity in µM range), rather than exhibiting agonistic activities [33]. However, additional studies are necessary to confirm this hypothesis and to demonstrate the preciese mechanism though which scutigeral interacts with the TRPV1 channel. 

### Other TRPV1 Modulators

Camphor is isolated from the wood of the camphor laurel tree (*Cinnamomum camphora*) and is commonly applied to the skin for antipruritic, analgesic, and counterirritant properties [12]. Recently, it was shown that camphor is able to activate heterologously expressed TRPV1, requiring higher concentrations than capsaicin. Activation was enhanced by phospholipase C-coupled receptor stimulation mimicking inflamed conditions. Camphor activation of rat TRPV1 was mediated by distinct channel regions from capsaicin, as indicated by camphor activation in the presence of the competitive inhibitor capsazepine. Although camphor activates TRPV1 less effectively, camphor application desensitized TRPV1 more rapidly and completely than capsaicin. However, camphor appears to act as a nonselective TRPV1 agonist because it is also capable to activate other TRP channels, see later [134]. 

Recently, evidence was showed for desensitization-dependent TRPV1 activation in cnidarian envenomations [23]. Cnidarian venom induced reactivity cause erythema, burning pain, hypersensitivity and inflammation [128]. Although none of the venom extracts of four cnidarian species (*A. pulchella*, *C. flockerzi*, *P. physalis* and *C. capillata*) had significant effect of its own, there was a clear allosteric effect on the activation of the channel when the venom was applied with capsaicin. These findings can suggest that the venom prevents the desensitization of TRPV1, which results in a larger inward current that would generate in turn, the typical persistent burning-pain sensation [23]. *In vivo* experiments showed that cnidarian venom induced a nociceptive reactivity, comparable to capsaicin, in laboratory rats, which could be reduced by the selective TRPV1 antagonist, N-(4-Tertiarybutylphenyl)-4(3-cholorphyridin-2-yl)-tetrahydro-pyrazine1(2H)-carboxamide (BCTC) [23]. The active substance(s) in these venoms is (are) not identified (i.e. whether peptide or bioactive small molecule) and the precise site of interaction still remains to be elucidated (i.e. on the TRPV1 itself *vs*. the signal-transduction pathway). Similarly, the same group also reported that gambierol and brevetoxin increase currents through pre-activated TRPV1 channels. These compounds are polyether toxins which are believed to underlie ciguatera fish poisoning and neurotoxic shell fish poisoning, resulting from the consumption of tropical reef fishes and shell fish [24]. 

Siemens *et al*. showed that venom from the tarantula, *Psalmopoeus cambridgei* that is native to the West Indies, contains three peptides that target TRPV1 [99]. Using calcium imaging and further purification a family of three closely related peptides was revealed, i.e. vanillotoxins (VaTx) 1, 2 and 3. Vanillotoxins are new members of the extended family of inhibitor cysteine knot (ICK) peptides from spiders and cone snails [142]. ICK toxins are widely recognized as blockers of cationic channels, perhaps best characterized by the interaction of hanatoxins with voltage-gated (K_v_) potassium channels [107, 108]. In contrast with the predominant role of ICK toxins as channel inhibitors, these unknown vanillotoxins function as TRPV1 agonists. Capsaicin and vanillotoxins share similarities in their mechanisms of TRPV1 activation (voltage-dependency), but probably interact with distinct regions of the channel. The toxin elicited responses in outside-out patches only, whereas capsaicin was effective in either configuration [99]. *In vivo* experiments in wild-type mice showed that injection of capsaicin into the hind paw elicits pain-related behaviors, such as licking and flinching of the affected limb. Injection of purified VaTx3 produced similar responses in wild-type mice, but not in TRPV1-deficient littermates. Also the hind paws of toxin-injected wild-type mice showed substantial oedema, whereas TRPV1-deficient animals displayed only minimal swelling akin to that observed with vehicle-injected wild-type controls [99]. 

Porcine TRPV1 can be activated by allyl-isothiocyanate, the main pungent compound of mustard oil [87]. This is a remarkable finding since data from TRPA1 KO mice indicated that only TRPA1 is mediating the pungent effect of mustard oil in mice (for a more detailed discussion, see below). Whether this is a species dependent effect is unknown at the moment.

### TRPV1 Inhibitors

Thapsigargin, is the active ingredient of the Mediterranean plant *Thapsia garganica*, which was used in traditional European and Arabian medicines for rheumatic pain. Like the ultrapotent vanilloid analog resiniferatoxin, thapsigargin is a natural product based on a tricyclic diterpene ring. Studies have shown that thapsigargin inhibited TRPV1 mediated [^45^Ca^2+^] -uptake and blocked [^3^H]-RTX binding sites in rTRPV1-CHO cells. However, thapsigargin has higher affinity for SERCAs (nanomolar range) than for TRPV1 [120].

Yohimbine, an indole alkaloid obtained either from the bark of the tree *Pausinystalia yohimbe*or the root of *Rauwolfia.* It was initially identified as an aphrodisiac, and later introduced in the treatment of erectile dysfunction. Yohimbine is know as a natural (-adrenoreceptor antagonist [62] and is frequently used to characterize the mechanism of a drug’s action on (-adrenoreceptors. Dessaint *et al*. showed that yohimbine inhibit Na^+^ channels and TRPV1 channels in a dose dependent way. Action potential firing activities of DRG neurons by current injection or capsaicin were eliminated by yohimbine [28], further indicating that yohimbine is able to interfere with pain sensation or transduction. 

Robust inhibitory activity was found in venom from a North American funnel web spider, *Agelenopsis aperta*. Fractionation of the venom resulted in the purification of two acylpolyamine toxins, AG489 and AG505, which inhibit TRPV1 channels from the extracellular side of the membrane. The toxin AG489 was found to inhibit TRPV1 in a voltage-dependent way, with relief inhibition at positive voltages, consistent with the toxin inhibiting the channel through a pore-blocking mechanism [48]. 

## TRPV2

TRPV2 has also been proposed as a potential pain target, in part due to its sequence similarity to TRPV1 and due to its reported activation by noxious high temperatures (>52°C) [1, 16, 118]. Very recently, a first natural modulator for TRPV2 was identified, Δ9-tetrahydrocannabinol (THC), the main psychoactive substance found in the Cannabis plant. THC's most likely function in Cannabis is to protect the plant from herbivores or pathogens. THC also possesses high UV-B (280-315 nm) absorption properties, protecting the plant from harmful radiation. Both rat and human TRPV2 could be activated by THC in a dose-dependent way, and blocked by ruthenium red [79]. 

## TRPV3

TRPV3 was cloned by using its sequence homology to other heat-activated TRP channels, and shares 40% identity with TRPV1 [136]. TRPV3 exhibit thresholds in the physiological temperatures range of 32 to 39°C and can be activated by 2-APB, [19]. The warm-sensitive ion channel, TRPV3, which is highly expressed in the skin, tongue and nose [135], is a target for the flavor actions of several plants as well as for skin sensitization. The natural compound camphor (*Cinnamomum camphora* tree, (see above)), which modulates sensations of warmth in humans [29], proved to be an activator of TRPV3 [73].

Furthermore, TRPV3 is strongly activated and sensitized by other monoterpenes like carvacrol, carveol, thymol and (+)-borneol [125]. Terpenoids have long been recognized as medically and pharmacologically active compounds. Carvacrol, the major ingredient of oregano (*Origanum majorana*/ *vulgare*), and thymol, a lesser component of oregano but an important constituent of thyme (*Thymus vulgaris*) are both known to evoke a sense of warmth and sensitize skin [135]. Vanillin, the active ingredient of vanilla (*Vanilla planifolia*), weakly activates TRPV3, whereas the more potent synthetic vanillin much more potently activates TRPV3. Other natural compounds, like eugenol also activate and sensitize TRPV3. Eugenol, the principal ingredient of clove, from the *Caryophyllus aromaticus* tree, is used in dentistry as a topical analgestic [135]. The best agonists for TRPV3 were found among the monocyclic monoterpenes with a cyclic structure and a hydroxyl group [125]. Also menthol, a compound obtained from the oil of peppermint (*Mentha piperita*), popularly known for its cooling effect, activates heat-activated TRPV3 [65]. At warm temperatures menthol might be interpreted as warm based on its sensitizing effect on TRPV3, while at cooler temperatures, its activation of TRPM8 dominates its sensory quality [65]. 

## TRPV4

TRPV4 can be activated by physical stimuli (cell swelling, sheared stress and moderate warmth (>27°C) [31, 56, 83, 85, 105, 130, 132]), by the synthetic phorbol ester 4(-phorbol 12,13-didecanoate (4α-PDD) [129], and by epoxyeicosatrienoic acids derived from arachidonic acid [85, 130]. Very recently, a new TRPV4 agonist was identified. An extract from the plant *Andrographis paniculata* potently activate TRPV4 channels. This plant is used in traditional medicine in various parts of Asia for a wide array of ailmants. Extracts are typically used as an anti-inflammatory agent or immunostimulant; however, there have been a small number of studies on the cardiovascular effects of *Andrographis* [140]. The plant extract was fractionated further, and the active compound was identified as bisandrographolide A. This compound activates specifically TRPV4 channels [100]. In a latest study, evidence was provided that the TM3-4 region of TRPV4 forms an important site for channel activation by phorbol esters and bisandrographolide A. In particular, mutations at positions Leu^584^ and Trp^586^ in TM4 strongly affect channel activation by the most potent and selective agonists 4(-PDD and BAA [126].

## TRPM CHANNELS

The TRPM subfamily consists of 8 members, which are sub-divided in three groups on the basis of sequence homology: TRPM1/3, TRPM4/5 and TRPM6/7, with TRPM2 and TRPM8 being distinct proteins [81]. Three members of this subfamily are carrying an entire functional enzyme in their COOH-termini: TRPM2 contains a functional NUDT9 homology domain, exhibiting ADP-ribose pyrophosphatase activity, whereas both TRPM6 and TRPM7 contain a functional COOH-terminal serine/threonine kinase. Except for TRPM1 which is not functionally characterized until now, all TRPM channels are cation channels, although the Ca^2+^ permeability is diverse, ranging from highly permeable (TRPM6/7 and splice variants of TRPM3) to Ca^2+^ impermeable (TRPM4/5). TRPM4 and TRPM5 are heat-sensitive, Ca^2+^ activated cation channels. TRPM2 is activated by intracellular ADP-ribose, hydrogen-peroxide and heat. TRPM3 channels are, much like TRPM6 and TRPM7, regulated by intracellular Mg^2+^ levels. They show considerable constitutive activity, though TRPM3 activation was also reported after cell swelling and by sphingosine. TRPM8 is the infamous cold receptor [30, 38, 52, 68, 76, 82, 91, 92, 97, 117]. To date only TRPM3 and TRPM8 have been reported to be directly modulated by natural products.

## TRPM8

TRPM8 was originally identified as a prostate specific gene, up-regulated in malignant cancer [121]. Further research has shown that TRPM8 is also expressed in a subset of sensory neurons from DRG and trigeminal ganglia [68, 78, 91], in nodose ganglion cells innervating the upper gut [141], gastric fundus [75], vascular smooth muscle [138], liver [34], and in bladder urothelium and different tissues of the male genital tract [102]. In sensory neurons TRPM8 probably functions as a cold-thermo-sensor. Overexpressed in HEK293 cells, TRPM8 has been extensively characterized as a voltage-dependent, non-selective cation channel, which can be activated by cold temperatures (<25^°^C) and various cool compounds such as menthol. Apart from it’s activation by cold temperatures, a body of literature has reported channel TRPM8 activation by natural compounds [68, 91, 124].

### TRPM8 and Monoterpenes

The most potent natural compound activating TRPM8 is menthol, commonly known for it’s cooling sensation when eaten, inhaled or applied to the skin. Menthol is a natural monoterpenoid synthesized in plants from the *Mentha* genus [13]. In HEK293 and CHO cells overexpressing TRPM8, menthol application dose-dependently increases intracellular Ca^2+^. Patch-clamp experiments revealed activation of a strongly outwardly rectifying, Ca^2+^ permeable cation current in TRPM8 overexpressing but not control cells, with strong similarities to the endogenous cold- and menthol-activated current in trigeminal neurons [68, 91]. An elegant analysis of the voltage dependence of TRPM8 revealed that menthol and cold actually use the same mechanism to activate the TRPM8 channel, i.e. through shifting the voltage-dependent activation curve of TRPM8 to more physiological potentials [122]. Concomitantly, mutation of voltage sensing residues in the transmembrane domain 4 (TM4) and the TM4-TM5 linker similarly influenced menthol- and cold-sensitivity of TRPM8 [123]. However, this does not imply that menthol and cold-sensitivity of TRPM8 are necessarily linked. An extensive mutagenesis study revealed that mutation of specific residues in transmembrane region 2 and the C-terminus of the protein of TRPM8, resulted in an menthol-insensitive cold-sensitive channel. Specifically the critical residues in TM 2 are suspected to contribute to the menthol binding site of TRPM8 [5].

Other monoterpenes that activate TRPM8 are eucalyptol (present in essential oils from *Eucalyptus polybractea*), menthone (the precursor of menthol in monoterpene biosynthesis), geraniol (found in lemon-grass and aromatic herb oils), linalool found in floral scents of Onagraceae species), menthyl lactate (from peppermint oil), trans- and cis-p-menthane-3,8-diol from *E. citriodora*), L-carvone (from spearmint or Kuromoji oil), isopulegol (from *M. pulegium* or *Lilium ledebourri*) and hydroxyl-citronellal (from citronella oils, volatile oils such as lemon, lemongrass or melissa oils) [8, 68]. Camphor or cyclohexanol have no effect [68]. In contrast to menthol, the activating effect of these compounds is less characterized. It is unclear whether these compounds use the same binding site as menthol on the TRPM8 protein, or whether they bind to the channel at all.

### Other Modulators of TRPM8: Eugenol and Ethanol

Apart from the above mentioned monoterpenoid compounds, a FLIPR study revealed that TRPM8 is also activated by eugenol (from clove oil, see above [8]). However, this compound also activates other TRP channels, including TRPV1, TRPV3 and TRPA1.

Ethanol, on the other hand, inhibits TRPM8 by modulating it’s interaction with membrane phosphatidylinositol 4,5-bisphosphate (PIP_2_). Activation of TRPM8 by cold or menthol is followed by significant channel desensitisation [68, 91], and this desensitization depends on the presence of Ca^2+^ in the extracellular medium [68]. Recently, it was shown that phosphatidylinositol 4,5-bisphosphate (PIP_2_) plays a key-role in this process. PIP_2_ acts as a positive modulator of the channel’s sensitivity for cold or menthol and prevent channel rundown in cell-free patches, most likely by shifting the voltage-sensitivity of activation towards physiological voltages [57, 96]. Thus, application of PIP_2_ alone to excised membrane patches activates the channel. As for the desensitization process, Rohacs and colleagues proposed that Ca^2+^ influx through TRPM8 leads to activation of Ca^2+^-dependent phospholipase C (e.g. PLCδ1), leading to depletion of cellular PIP_2_ levels and channel closure [96]. They also identified positively charged residues in the TRP domain, which are located C-terminal of TM6, and which participate in the interaction of the channel with membrane-bound PIP_2_ [96].

Ethanol inhibits TRPM8 activity. This occurs presumably by interfering with the TRPM8 PIP_2_ interaction, since inclusion of PIP_2_ in the patch-pipette results in a strong reduction of the ethanol-induced inhibition of menthol-evoked processes. Of note, ethanol potentiates the activity of the heat- and capsaicin-gated vanilloid receptor TRPV1 [9]. 

## TRPM3

TRPM3 transcripts are expressed in kidney (though disputed), eye and brain, in the latter specifically in regions such as the dentate gyrus, the intermediate lateralseptal nuclei, the indusium griseum, and the tenia tecta. Strongest *Trpm3* expression was found in the epithelialcells of choroid plexus [86]. Studies on the functional role of TRPM3 are fairly complicated by the fact that the TRPM3 gene is alternatively spliced into multiple functional variants. To date it was reported that at least TRPM3α1 and TRPM3α2 form cation-selective channels, though only TRPM3α2 is a Ca^2+^ permeable channel. Both exhibit constitutively active, outwardly rectifying currents that are blocked by intracellular Mg^2+^ [86]. Interestingly, TRPM3α2 is reported to be activated by some naturally occurring steroidal compounds, unlike TRPM3(1, but further information is lacking at the time of writing [72, 80]. Another splice variant, the 1325 amino acid variant of TRPM3 is activated by the sphingolipid D-*erythro*-sphingosine (SPH) and by SPH analogs, but not sphingosine-1-phosphate or ceramide. SPH is a central metabolite arising during the de novo synthesis of cellular sphingolipids [30]. Further insight in the physiological importance of these activating compounds will inevitably require elucidation of the expression of specific splice variants in specific tissues, an issue that is highly unclear at the moment.

## TRPA1

TRPA1 (before ANKTM1) is another member of the TRP family which has attracted wide attention in the field of sensory physiology. TRPA1 is distantly related to the other members of the TRP family, but shares the basic architecture with 6 transmembrane (TM) domains and a pore region between TM5 and 6. Strikingly, the C-terminus of TRPA1 contains as many as 14 ankyrin binding domains. In the first report, TRPA1 was described as a protein interfering with normal cell growth [44]. In subsequent papers, TRPA1 turned out to be a Ca^2+^ permeable cation channel, activated by cold temperatures (<17^°^C), provoking the hypothesis that TRPA1 functions as a detector of noxious cold *in vivo* (Story *et al*., 2003). TRPA1 is expressed in small-diameter neurons of the trigeminal and dorsal root ganglia, but apart from that also in hair cell epithelium. Cold activation of TRPA1 is however a disputed issue [45, 104] and the question whether TRPA1 functions as a cold detector remains unresolved even after analysis of two independent knockout mouse lines [7, 51]. More interesting in the context of this review, however, it is clear that TRPA1 is activated by an intriguing set of natural products, including pungent chemicals as allyl isothiocyanate (mustard oil), allicin (from garlic), cinnamaldehyde (from cinnamon), methylsalicylate (winter-green), eugenol (cloves) and gingerol (ginger).

### Isothiocyanate Compounds and Cinnamaldehyde

Isothiocyante derivatives constitute the main pungent ingredients in wasabi (allyl isothiocyanate), yellow mustard (benzyl isothiocyanate), Brussels sprouts (phenylethyl isothiocyanate), nasturtium seeds (isopropyl isothiocyanate) and capers (methyl isothiocyanate). Allyl isothiocyanate is the major active ingredient in mustard oil. Topical application of mustard oil to the skin activates underlying sensory nerve endings, thereby producing burning pain, inflammation and robust hypersensitivity to both thermal and mechanical stimuli [13], but the mechanism through which these compounds elicit their effects was unknown until recently. Calcium imaging and electrophysiological analysis showed that each of the above mentioned compounds was capable of activating human TRPA1, expressed in oocytes [45]. Concomitantly, allyl thioisocyanate elicits a Ca^2+^ response in subset of cultured neurons trigeminal and dorsal root ganglia, which is dependent on extracellular Ca^2+^, indicating that a Ca^2+^ influx channel is involved [45, 104]. Importantly, this response is completely lacking in *Trpa1*^–/–^ mice [7], indicating that TRPA1 is the sole target through which mustard oil activates primary afferent nociceptors. Notably in this regard however, porcine TRPV1 can also be activated by allylisothiocyanate [87]. Whether this is a species dependent effect is unclear at the moment.

Cinnamaldehyde is the main constituent isolated from cinnamon oil which is the essential oil obtained from *Cinnamomum cassia* or *Cinnamomum zeylanicum *[13]. It is routinely used for flavouring purposes, and in human subjects, cinnamaldehyde is reported to elicit a burning and tingling sensation in the mouth. When TRPA1 is overexpressed in CHO cells, it can be activated by micromolar concentrations of cinnamaldehyde, that is, an increase in intracellular Ca^2+^ concentration was observed [6]. To date no current measurements for TRPA1 activated by cinnamaldhyde have been reported. Notably, no response was obvious when cinnamaldehyde was applied to TRPV1, TRPM8 and TRPV4 expressing cells [6, 45]. When injected, cinnamaldehyde elicits pain-related behavior in mice. As expected, this response is similar in *Trpv1*^–/–^ mice. Further testing in *Trpa1*^–/–^ mice has not yet been reported [6].

Intriguingly, when different cinnamaldehyde-related com-pounds were tested, cinnamic alcohol was less potent, and cinnamic acid not a TRPA1 activator at all [6]. This finding contained the clue to the elucidation of the activation mechanism of TRPA1 by cinnamaldehyde and isothiocyantates. Indeed, as [36] and [63] reported, several structurally distant TRPA1 activators are membrane permeable electrophiles, suggesting that covalent modification of thiols and primary amines, rather than classical lock-and-key agonist binding, accounts for the activating effects of these compounds. Thus, Macpherson *et al.* (2007) were able to design a more potent TRPA1 activator by modifying the cinnamaldehyde structure, making it more reactive towards the nucleophilic mercaptogroup of cysteines (super-cinnamaldehyde). Hinman *et al.* could identify three cysteine residues in the cytoplasmic N-terminus of the channel which are essential for activation of TRPA1 by thiol reactive compounds. MacPheron *et al.* on the other could not confirm two of these three cysteine residues, but instead found two others, and additionally could show that all three are indeed covalently modified by mustard oil and super-cinnamaldehyde. Therefore, the exact site of chemical modification of TRPA1 remains unclear at the moment. Notably, activation of TRPA1 by other means such as cold, voltage and PLC-mediated activation was not affected by mutating these residues [36, 63].

### Other Natural Products

CHO cells overexpressing TRPA1 show a sharp increase in intracellular free Ca^2+^ levels when stimulated with plant-derived compounds such as eugenol (from clove oil), gingerol (from ginger) and methyl salicilate (from wintergreen oil) [6]. All these compounds cause a pungent burning sensation in humans. Whether these effects are mediated solely through TRPA1 activation remains unclear, since both TRPM8 and TRPV1 are also activated by these compounds. Likely, the *Trpa1*^–/–^ mouse models will be helpful in resolving this issue. Both TRPA1 and TRPV1 are activated by extracts from raw but not baked garlic extracts. More specifically, allicin, an unstable compound of fresh garlic, is the chemical responsible for TRPA1 and TRPV1 activation [64]. It is commonly known that raw garlic elicits burning pain and prickling sensations on the lips and the tongue. Trigeminal and dorsal root ganglion neurons from *Trpa1*^–/–^ mice were completely insensitive to allicin, indicating that TRPA1 is the sole site of action of these compounds. Another plant-derived compound activating TRPA1 is carvacrol, the major ingredient of oregano. TRPA1 is rapidly activated and desensitized by this compound, as is however also TRPV3 [135]. On the contrary, basal activity of TRPA1 at room-temperature is blocked by camphor [134]. Camphor is a plant-derived product (see above), and activates both TRPV3 and TRPV1. The camphor-induced desensitization of TRPV1 and block of TRPA1 might underlie the analgesic effects of camphor [134].

In contrast to TRPM8, TRPA1 is blocked by menthol. That is, 250μM menthol significantly inhibits both the cold-induced rise of [Ca^2+^]_cyt_ as cold-induced currents in TRPA1 transfected CHO cells [65]. As mentioned above however, cold activation of TRPA1 is a matter of intense debate. In a recent report, it was claimed that TRPA1 is indirectly activated by a cold-induced rise of [Ca^2+^]_cyt_, i.e. that TRPA1 is actually a Ca^2+^ activated channel [143]. The selective activation of TRPA1 and TRPM8 by mustard oil and menthol is an important topic, since it is used as a tool for the identification of distinct populations of sensory neurons from trigeminal or dorsal root ganglia. This has led to conflicting results in a sense that *in situ* hybridization and immunostaining studies have shown that TRPM8 and TRPA1 are expressed in a mutually exclusive fashion, while, on the other hand, functional studies have shown menthol responses in a significant (but highly variable) fraction of mustard oil or cinnamaldehyde responsive neurons [4, 45]. Several issues could hamper these results, including the selectivity of the *in situ* hybridization strategy and the antibodies used for immunostaining. But more importantly, also the selectivity of the activating compounds could be questionable. The respective knock-out mouse models will be indispensable to resolve whether menthol and mustard oil or cinnamaldehyde are really selective agonists for TRPM8 and TRPA1, respectively.

Intriguingly, TRPA1 is also activated by Δ9-tetrahydro-cannabinol (THC) and cannabinol (an oxidation product of THC). THC is the major psychoactive cannabinoid in the Cannabis species. Both in TRPA1 overexpressing CHO cells as in trigeminal neurons, THC and cannibinol activate TRPA1 [45]. When consumed by humans, THC produces a wide range of biological effects, such as an increase in pulse rate, decreased blood-pressure, muscle weakening, increased appetite, and euphoria, followed by drowsiness. Most of the effects of THC have been related to activation of cannabinoid receptors [37], but a recent study documented relaxation of hepatic or mesenteric arteries *in vitro* independent of cannabinoid receptors, by a mechanism involving activation of capsaicin-sensitive and CGRP- containing perivascular sensory nerve endings that innervate the smooth muscle [144]. Thus, the possibility remains that some of the biological effects of THC can be attributed to TRPA1 activation. The physiological relevance of this will certainly be clarified using the *Trpa1*^–/–^ mouse.

## TRPP

Among the TRP subfamilies, the polycystic kidney disease (PKD) family, also called polycystins or TRPP, is a relative newcomer [27]. The TRPP family is structurally divided into two groups, polycystic kidney disease 1-like proteins (PKD1-like, TRPP1-like), including PKD1, PKDL1-3 and PKDREJ, and polycystic kidney disease 2-like (PKD2-like, TRPP2-like) proteins, including PKD2, PKD2L1 and PKD2L2, which have a completely different protein architecture. TRPP1-like proteins consists of 11 transmembrane domains, a very long (2500aa) and complex extracellular domain and a C-terminal domain which interacts with C-terminus of TRPP2. The latter has a more classical TRP architecture with 6 TM domains although the pore-region is presumably located between TM1 and TM2 [27]. The functional characterization of these proteins is less developed, compared to other members of the TRP family. 

TRPP1 and TRPP2 were originally identified during the study of autosomal dominant polycystic kidney disease, an inherited disorder which is the most common cause of renal failure in humans. Mutations in the gene product of PKD1 account for roughly 85% of all cases of this severe disease condition, the remaining 15% being attributed to mutations in the PKD2 gene [40]. There is considerable evidence that TRPP1 and TRPP2 physically couple to function as a signaling complex. TRPP1 binds to and activates various G-proteins, and is also linked to other signaling pathways including the activation of the Janus kinase 2/signal transduction activating transcription 1 pathway, and nuclear translocation of NFAT transcription factor. PKD2 on the other hand is a Ca^2+^ permeable cation channel, of which the subcellular localization is highly controversial. Studies have located the channel both in the plasma-membrane and the endoplasmic reticulum [27]. In ER membrane, the channel would function as Ca^2+^ release channel, appearing to be directly activated by Ca^2+^. In the plasma-membrane it constitutes a constitutively open cation channel. The association of TRPP1 and TRPP2 suppresses the ability of TRPP1 to activate G proteins as well as the constitutive channel activity of TRPP2 [81]. 

A recent report shows that triptolide, the active diterpene in the traditional Chinese medicine Lei Gong Ten, binds to TRPP2 directly and induces Ca^2+^ release by a TRPP2-dependent mechanism. Triptolide is extracted from the plant *Tripterygium wilfordii, *which is a vine used in traditional Chinese medicine for the treatment of fever, edema and carbuncle, and for its male anti-fertility effect [93]. In murine model of autosomal dominant polycystic kidney disease, triptolide arrests cellular proliferation and attenuates cyst formation an effect that is largely attenuated in Trpp2P^–/–P^ mice [54]. 

On the other hand, it was shown that PKD2L1 in the tongue is expressed in a subset of taste receptor cells distinct from those responsible for sweet, bitter and umami taste [60]. Furthermore, mice lacking PKD2L1 expressing receptor cells are completely devoid of taste responses to sour stimuli [39]. In a different study it was reported that in fact PKD1L3 and PKD2L1 are both necessary to form a functional sour receptor [42]. When either protein was expressed alone in HEK293 cells, no sour responses are apparent, but when both proteins are expressed together HEK293 cells respond to HCl, malic acid and citric acid with an increase of the intracellular Ca^2+^, indicating that PKD1L3 and PKD2L1 together form a sour activated Ca^2+^ permeable ion channel. Since citric acid is apparently a more potent activator of the channel complex compared to HCl, it might be suggested that not merely the proton concentration activates this receptor, but that specific interactions with natural products such as citric acid are also necessary [42]. 

## CONCLUDING REMARKS

Animals evolved chemosensory mechanisms in skin, tongue, nose and mouth to test, or to remember, their tolerance of substances before ingestion. Bites and stings from venomous creatures produce pain and inflammation as part of their defensive strategy to ward off predators or competitors. In turn, plants evolved chemicals to repel animals to avoid being eaten or attract them in order to spread their seeds. Indeed, the seed-bearing organs of plants, their fruits, are often endowed with substances that attract animals. In contrast, many leaves and other plant parts contain noxious substances that induce pain or allergic reactions. 

Ion channels of the TRP family form an important aspect of the sensory mechanism of animals throughout evolution. Indeed it has been shown that several TRP channels react to thermal, mechanical and/or painful stimuli, and are at the same time modulated by natural compounds which can elicit a certain sensory sensation. Menthol for instance activates the cold-activated ion channel TRPM8 and thus tastes cool. Plants have an almost limitless ability to synthesize aromatic substances, most of which are phenols or their oxygen-substituted derivatives, such as tannins. To date at least 12,000 compounds have been isolated, a number estimated to be less than 10% of the total. In many cases, these substances (esp. alkaloids) serve as plant defense mechanisms against pre-dation by microorganisms, insects, and herbivores. The search for the molecular targets for naturally occurring substances, allowed the characterization of many TRP channels. In fact, attempts to understand the hot and painful action of the vanillyl group containing compounds capsaicin (from *Capsicum* sp.) and its ultrapotent analogue resiniferatoxin (from *Euphorbia* sp.) led to the cloning of TRPV1, and the development of a whole new generation of painkillers [113].

Species differences for a specific channel gene can have intriguing consequences. Birds, toads, and reptiles are insensitive to the pain-producing effects of capsaicin [46, 116]. However, birds exhibit heat-evoked membrane currents resembling those carried by the mammalian TRPV1. These observations suggest that birds express a vanilloid-insensitive homologue of the mammal TRPV1, and indeed, it was shown that small species differences in coding lead to the fact that the chicken receptor is activated by heat or protons but is insensitive to capsaicin. As a result, birds are better *Capsicium* seed dispensers than mammals [46]. Similar observations have also shown differences between the rat and human isoform with regard to sensitivity to agonist binding, with the rat receptor binding resiniferatoxin at a higher affinity than the human [18]. 

The use of and search for drugs and dietary supplements derived from plants has accelerated in recent years. Pharmacologists, microbiologists, botanists, and natural-products chemists are combing the Earth for phytochemicals and lead-structures that could start-off new treatments of various diseases. In this review, we clearly illustrate that many natural compounds modulate TRP channels. However, a global analysis of the available data in the literature clearly shows that the knowledge of the physiological role of TRP channels is just in its infancy. Thus, the consequences and clinical relevance of recent findings can be hard to anti-cipate. However, it is clear that at least some practices of traditional plant medicine can be nicely explained since the discovery of TRP channels. Finally, it is obvious from this review that naturally occurring substances, especially those derived from higher plants, will continue to be essential tools in the discovery of therapeutic targets necessary for the development of new innovative drugs.

## Figures and Tables

**Table 1.TRPV1:** 

Compound	Structure	Origin	Action	Conc. Range
Capsaicin	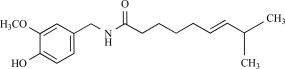	*Capsicum *genus	Activates (17)	10^-8^-10^-6^M
Piperine	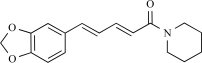	*Piper nigrum/ longum*	Activates (69)	10^-5^-10^-4 ^M
Eugenol	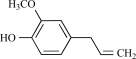	Clove oil	Activates (137)	10^-3^-10^-2 ^M
Resiniferatoxin	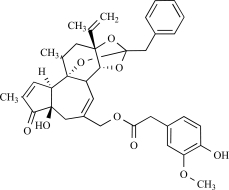	*Euphorbia resinifera*	Activates (17)	10^-10^-10^-8 ^M
Gingerol	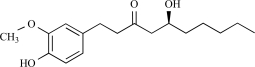	*Zingiber officinale*	Activates (43)	10^-4 ^M
Zingerone	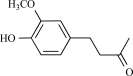	*Zingiber officinale*	Activates (59)	10^-2 ^M
Evodiamine	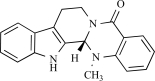	*Evodia rutaecarpa*	Activates (89)	10^-6^-10^-5^M
Cannabidiol	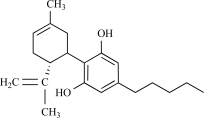	*Cannabis sativa*	Activates (10)	10^-6^-10^-5 ^M
Polygodial	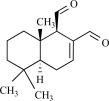	*Polygonum hydropiper*	Activates (2)	10^-6^-10^-5 ^M
Isovelleral	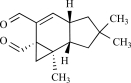	*Lactarius vellereus*	Activates (94, 114)	
Camphor	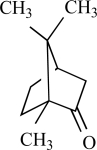	*Cinnamomum camphora*	Activates (134)	10^-3^-10^-2 ^M
Vanillotoxin 1, 2 and 3		*Psalmopoeus cambridgei*	Activates (99)	10^-8^-10^-4 ^M
Cnidarian envenomations		jellyfish	Activates (23)	
Thapsigargin	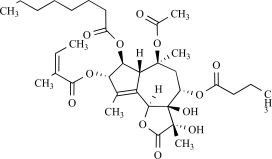	*Thapsia garganica*	Inhibits (120)	10^-6^-10^-5 ^M
Yohimbine		*Pausinystalia yohimbe*	Inhibits (28)	10^-5^-10^-4 ^M
AG489 toxin	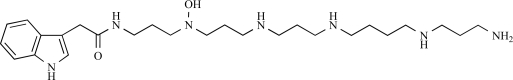	*Agelenopsis aperta*	Inhibits (48)	10^-7 ^M
AG505 toxin	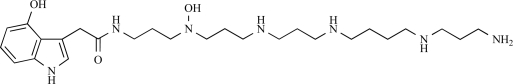	*Agelenopsis aperta*	Inhibits (48)	10^-7^M

**Table 2. Other TRPVs:** 

Compound	Structure	Origin	Action	Conc. Range
Carvacrol	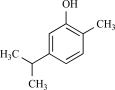	*Origanum*genus	Activates TRPV3 (124)	10^-4^-10^-3 ^M
Eugenol	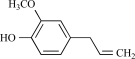	Clove oil	Activates TRPV3 non specific (124)	10^-5^-10^-3 ^M
Thymol	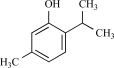	*Thymus vulgaris*	Activates TRPV3 non specific (124)	10^-4 ^M
Camphor	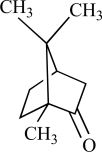	*Cinnamomum camphora*	Activates TRPV3 non specific(73)	10^-4^-10^-2 ^M
Vanillin	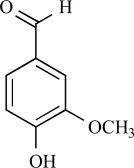	*Vanilla planifolia*	Activates TRPV3 (124)	
Menthol	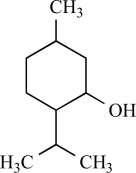	*Mentha *genus	Activates TRPV3 non specific (65)	10^-4^-10^-3 ^M
Bisandrographolide A	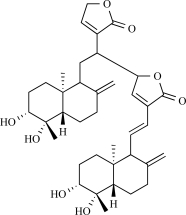	*Andrographis paniculata*	Activates TRPV4 (99)	10^-7^-10^-6 ^M

**Table 3. TRPM8:** 

Compound	Structure	Origin	Action	Conc. Range
Menthol	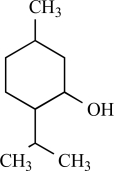	*Mentha *genus	Activates (68, 91)	10^-5 ^- 10^-3^ M
Eucalyptol	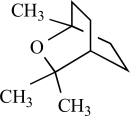	*Eucalyptus polybractea*	Activates (8)	10^-4 ^- 10^-2^ M
Menthone	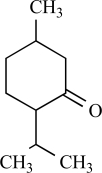	Precursor in menthol biosynthesis	Activates (68)	10^-4 ^- 10^-3 ^M
Geraniol	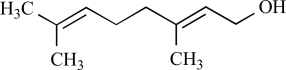	*Cymbopogon nardus*	Activates (8)	10^-2 ^- 10^-3 ^M
Linalool	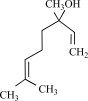	*Onagraceae* genus	Activates (8)	10^-2 ^- 10^-3 ^M
Menthyl lactate	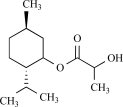	Peppermint oil	Activates (6)	10^-4 ^- 10^-3 ^M
Cis- and trans-p-menthane3,8-diol	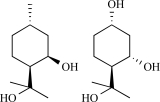	*Eucalyptus citriodora*	Activates (6)	10^-4 ^- 10^-3 ^M
L-carvone	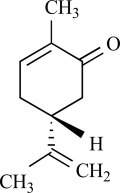	*Mentha spicata*	Activates (6)	10^-4 ^- 10^-3^M
Isopulegol	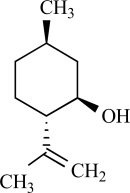	*Mentha pulegium * or *Lelium ledebourii*	Activates (6)	10^-4 ^- 10^-3 ^M
Hydroxyl-citronellal	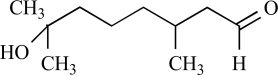	Citronella oil	Activates(8)	10^-2 ^-10^-3^M
Eugenol	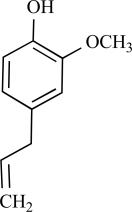	Clove oil	Activates (8) but also TRPA1 (6) and TRPV1 (137)	10^-3^ – 10^-4 ^M
Cinnamaldehyde	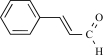	*Cinnamomum cassia* or *Cinnamomum zeylanicum*	Inhibits (65)	10^-2 ^- 10^-3 ^M
Ethanol	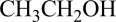		Inhibits by modulating PiP2 interaction (9)	0.1 – 1 M

**Table 4. TRPA1:** 

Compound	Structure	Origin	Action	Conc. Range
Allyl-isothiocyanate	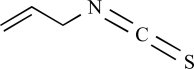	Wasabi, mustard oil	Activates (45)	10^-5^ – 10^-6 ^M
Benzyl- isothiocyanate	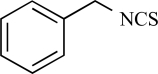	Yellow mustard	Activates (45)	10^-4^ – 10^-5 ^M
Phenylethyl- isothiocyanate	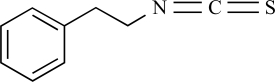	Brussels sprouts	Activates (45)	10^-4^ – 10^-5 ^M
Isopropyl- isothiocyanate	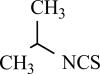	Nasturtium seeds	Activates (45)	10^-4^ – 10^-5 ^M
Methyl- isothiocyanate	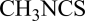	*Capparis spinosa*	Activates (45)	10^-4^ – 10^-5 ^M
Cinnamaldehyde	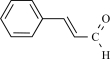	*Cinnamomum cassia* or *Cinnamomum zeylanicum*	activates (6)	10^-5^ – 10^-6 ^M
Eugenol	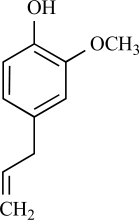	Clove oil	Activates (6) but also TRPM8 (8) and TRPV1 (137)	10^-3^ – 10^-4 ^M
Gingerol	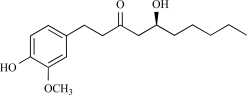	*Zingiber officinale*	Activates (6)	10^-3^ – 10^-4 ^M
Methyl salicilate	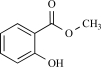	Wintergreen oil	Activates (6)	10^-3^ – 10^-4 ^M
Allicin	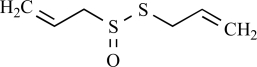	*Allium Sativum*	Activates but also TRPV1 (64)	10^-3^ – 10^-4 ^M
Carvacrol	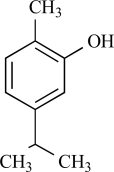	*Origanum* genus	Activates (135)	10^-3^ – 10^-4 ^M
Camphor	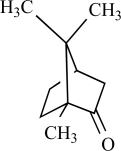	*Cinnamonum camphora*	Inhibits (134)	10^-2^ – 10^-3 ^M
Menthol	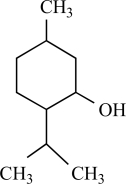	*Mentha *genus	Inhibits (65), but activates TRPM8 (68, 91)	10^-4^ – 10^-5 ^M
Δ^9^-tetrahydrocannabinol	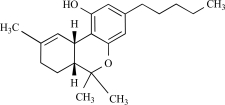	*Cannabis *genus	Activates (45)	10^-5^ – 10^-6 ^M
